# Computer-Based Collaborative Problem Solving in PISA 2015 and the Role of Personality

**DOI:** 10.3390/jintelligence7030015

**Published:** 2019-07-01

**Authors:** Matthias Stadler, Katharina Herborn, Maida Mustafić, Samuel Greiff

**Affiliations:** 1 Psychologie und Pädagogik, Ludwig-Maximilians-Universität München, 80802 Munich, Germany; 2 Computer Based Assessment, University of Luxembourg, Esch-Sur-Alzette, L-4366 Luxembourg, Luxembourg

**Keywords:** collaborative problem solving, PISA 2015, assessment, big five, personality, agent technologies

## Abstract

Collaborative problem solving (CPS) is an essential 21st century skill at the intersection of social collaboration and cognitive problem solving, and is increasingly integrated in educational programs, such as the influential Programme for International Student Assessment (PISA). As research has identified the impact of the Big Five personality traits either on cognitive ability or social collaboration skills in groups, this study firstly identified their impact on the conjoint construct of CPS. Results from structural equation modelling (*N* = 483) found openness to experience and agreeableness as predictors for CPS performance. The results are embedded in the lifelong learning and investment model by Ackermann and provide implications for PISA 2015, as original PISA 2015 CPS tasks were used.

## 1. Introduction

Problem solving skills in collaboration with others (i.e., collaborative problem solving (CPS)) are increasingly important in many aspects of life in the 21st century [1]. Especially in educational and professional settings, students regularly learn and work in groups and professionals collaborate with colleagues in order to share their expertise, divide tasks, and solve problems that cannot be addressed individually. Thus, CPS skills have evolved as essential domain-general skills for college and career readiness [2] and public integration in today’s society [3,4].

As an immediate consequence of the increasing relevance of CPS, national and international governmental education commissions and policymakers have recently started to integrate CPS into comprehensive educational initiatives and programs for students. Programs, such as the Partnership for 21st Century Learning (P21; [5]) or the International Assessment and Teaching of 21st Century Skills project (ATC21S; [1]), aim to foster and assess CPS to ensure sufficient CPS development during compulsory school education. Among these initiatives, the Programme for International Student Assessment (PISA; [6]) probably counts as the most influential, with a direct impact on educational policies. PISA assesses over 500,000 students from more than 70 countries every three years in the major scholastic domains of mathematics, science, and reading. Due to the increasing relevance of CPS as a 21st century skill, PISA began assessing CPS in 2015 as a transversal skill and just recently published the results on the country comparisons on CPS performance (for further information see [6]). Taking into consideration that CPS is increasingly required in virtual settings in our global and computerized society, PISA 2015 assessed CPS via computer-based CPS approaches to adequately prepare students for realistic future environments.

However, despite the increased relevance of CPS in general, and particularly within computer-based environments, academic research on CPS is currently scarce and far below what would be expected given the great current political and educational relevance of CPS. In fact, there is scant empirical evidence on how CPS relates to other constructs and how it can be generally predicted. However, CPS is defined as a skill at the intersection of social collaboration skills and cognitive problem solving skills, and an existing body of research has found that personality is a predictor for both of these constructs, both generally and in virtual environments. For example, research has found that individual group members’ Big Five traits (i.e., openness to experience, neuroticism, conscientiousness, extraversion, and agreeableness) are contributors to the resulting overall group performance [7], and that this finding can be generalized to virtual environments [8]. Interrelations have also been found between the Big Five personality traits and general cognitive ability [9], as well as cognitive problem solving in virtual tasks [10].

Investment theories, such as the lifelong learning and investment model (intelligence-as process, personality, interests, and knowledge; PPIK) by [11], ground these relations between cognitive ability and affective traits in their interdependent development during childhood and adolescence, which ultimately creates patterns of abilities, skills, personality, and interests [12]. The PPIK theory integrates intelligence-as-process, personality, interest, and intelligence-as-knowledge, thereby grounding ability levels and personality dispositions as determinates of success in particular task domains (e.g., science or mathematics) [13]. Transferring existing research and the PPIK theory onto CPS, the Big Five personality traits (i.e., openness to experience, neuroticism, conscientiousness, extraversion, and agreeableness) should also play a role in CPS to varying degrees, as CPS is a construct that integrates social collaboration and cognitive problem solving skills. However, existing findings investigate either the personality–social collaboration link or the personality–cognitive ability link separately, with no existing findings on the role of the Big Five in the conjoint construct of CPS. To better understand the nature of CPS and develop educational assessments and interventions, the role of personality in CPS needs to be explored.

To identify the role of personality in CPS, and address the discrepancy between a lack of academic research on the one hand and increasing educational and political demands on the other, this study first reviews the PISA 2015 CPS approach before subsequently investigating whether and to what extent personality can predict CPS performance. In a final step, as reasoning and reading skills play a role in CPS skills, we ensured that the association between personality and CPS is distinct by controlling for reasoning and reading performance.

## 2. Computer-Based Assessment of CPS in PISA 2015

In PISA 2015, CPS is defined as a skill “(…) to effectively engage in a process whereby two or more agents attempt to solve a problem by sharing the understanding and effort required to come to a solution and pooling their knowledge, skills and efforts to reach that solution” [14]. To assess CPS on the fine-grained level of specific aspects of cognitive problem solving in social collaborative environments, 12 distinguishable CPS skills were stipulated for PISA 2015. Each CPS skill represented a particular aspect of social collaboration with others (e.g., maintaining shared understanding within the group) or cognitive problem solving processes (e.g., planning and executing) (for further details on the 12 CPS skills, see the PISA 2015 “12-Cells Matrix” in Table 1, [14]). At the end of the PISA 2015 CPS assessment, CPS sub-skills were summed up to obtain an overall CPS score.

**Figure 1:**
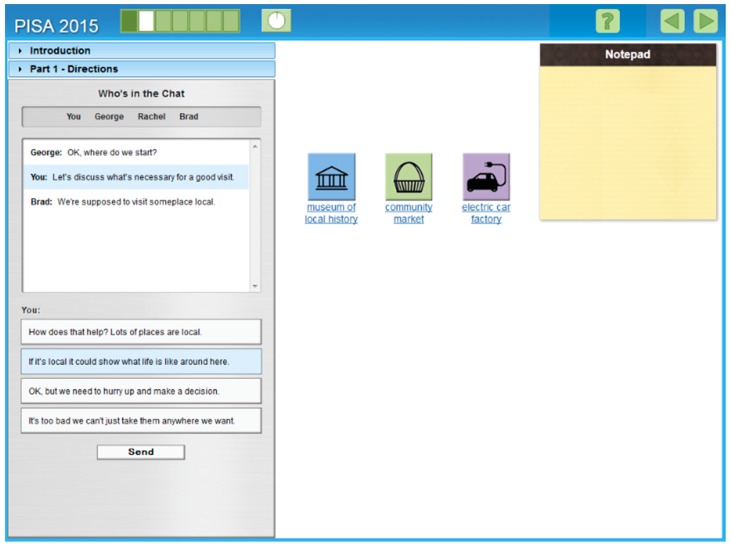
Screenshot of the released example PISA 2015 CPS task “The Visit” (OECD, 2015). The Visit is one officially published PISA 2015 CPS task. It only ran in the PISA 2015 field trial and not in the main study [15].

PISA 2015 chose a computer-based approach to assess students’ preparedness for present-day and future virtual CPS environments in our global and computerized 21st century society. More specifically, CPS skills in PISA 2015 were assessed via individual computer-based CPS tasks, which required the individual to collaborate with a minimum of one and a maximum of three virtual computer agents (human-agent (H-A)) approach in simulated real-life problem scenarios beyond the specific context of school subjects. Figure 1 depicts a screenshot of the example PISA 2015 CPS task “The Visit,” which required students to organize a trip for a school class. As shown in Figure 1, the English version of the PISA 2015 task “The Visit” required students to collaborate with the computer-simulated agents, George, Rachel, and Brad, by exchanging predefined messages in a chat box on the left side of the screen, while solving simulated problems in a task space on the right side of the screen (for more detailed information, see [14]). Students’ CPS skills were scored on the basis of their message selection and performance on problem solving actions; students always received a selection of predefined messages of which one (and in seldom cases two) was the correct answer, reflecting a specific CPS skill in this particular situation. For example, in Figure 1, the highlighted response is the correct answer and represents skill “B1,” as it helps to advance the group’s shared understanding of what “local” means (i.e., B1: Building a shared representation and negotiating the meaning of the problem, common ground) [15].

The general setup of the PISA 2015 CPS tasks was to simulate real-life collaborative environments by attributing varying characteristics and CPS skills to the agents, varying group sizes, and assigning different group roles to the students in the tasks. Therefore, the computer agents responded to the students’ requests differently across tasks, group sizes varied from two to four group members, and students performed different roles, such as coordinator or decision-maker, within the group. For example, in the example task, “The Visit,” Rachel was simulated as having stronger CPS skills than George and Brad by providing more helpful information, the group consisted of four members, and the student had to maintain shared understanding in the group. This enabled the simulation of real-life CPS environments and allowed CPS skills to be assessed in a standardized and controlled manner without external effects from collaboration partners, such as personality impacting the student data [14].

Whereas external effects on the assessed students can be controlled for via standardized assessment conditions, effects of students’ personality differences on resulting CPS performance are present, although their extent is unknown. While a growing body of research has found personality to be a predictor of both cognitive ability as well as social collaboration skills in groups, it can be hypothesized that personality also plays a role in CPS. However, the role of personality in the conjoint construct of CPS has not been investigated to date yet. Only selective aspects of CPS have been examined, and the role of individual personality in problem solving processes in social collaboration with others remains unclear. However, just as computer agents’ personalities change group interactions and CPS behaviors, the test-taker’s personality should also exert an effect on CPS performance within the group. Understanding personality differences in CPS performance could sharpen the development of CPS assessments and interventions. For example, findings on the role of personality for selective aspects of CPS, such as group composition [7], or how the combination of members’ personalities affects interaction and group performance [16] supported the development of CPS assessments, such as those for PISA 2015 (for further details, see [14]), which aim to sufficiently prepare students for current and future 21st century challenges. Therefore, we draw appropriate hypotheses from the separate bodies of literature on the personality–social collaboration link and the personality–cognitive ability link in our analysis of the role of personality in the conjoint construct of CPS.

## 3. The Triad between Personality and Social and Cognitive Abilities

### 3.1. Personality and Social Collaboration Abilities

Social collaboration represents an essential component in the conjoint construct of CPS, alongside cognitive problem solving, and personality research has identified personality traits as important influencers of individuals’ social collaboration behavior in groups. This collaborative behavior, in turn, shapes group members’ interactions and overall group performance. In fact, human resources departments within organizations are increasingly taking individual personality differences into account when composing groups of employees for work-related tasks [17]. The majority of existing academic research on the role of personality in social collaboration captures personality differences with the Big Five model, which summarizes personality according to the five main personality traits of openness to experience, neuroticism, conscientiousness, extraversion, and agreeableness (FFM; Big Five [18]). More specifically, a high level of openness to experience describes individuals with an active imagination (fantasy), aesthetic sensitivity, attentiveness to inner feelings, preference for variety, and intellectual curiosity [18]. In direct contrast, high levels of neuroticism characterize anxious, hostile, depressive, self-conscious, vulnerable, impulsive individuals who frequently experience negative moods [18]. Conscientious individuals are characterized as hardworking, responsible, self-disciplined, organized, and achievement-oriented [18], whereas extraverted individuals are described as sociable, outgoing, energetic, impulsive, and also less introspective [18]. High levels of agreeableness in individuals reflect trusting, frank, altruistic, cooperative, caring, and empathetic characteristics.

Meta-analytical results on the Big Five and their impact on social collaboration show that group members’ Big Five personality traits affect their social collaboration skills with other group members. For example, openness to experience reflects curiosity, preference for variety, and broad-mindedness, which seems to support social collaboration in groups. Homan and colleagues [16] found that open individuals encourage information exchange and the sharing of other group members’ ideas and opinions, enhancing overall group performance (for more specific meta-analytical results, see [19]). In direct contrast to this, high levels of neuroticism have been found to be a negative predictor of social collaboration, as neurotic individuals appear to be less cooperative, interact less with their fellow group members [20], and are less willing to help [21]. Therefore, groups with higher levels of neuroticism tend to perform lower in team performance tasks due to, for example, the development of negative work climates [22].

Furthermore, higher levels of conscientiousness are found among hardworking, organized, and achievement-oriented individuals, and tend to support social collaboration with others and thus group performance. Conscientious individuals enhance groups’ achievement orientation, and research has shown that groups with a high need for achievement outperform groups with a lower need for achievement in a variety of tasks [23]. Likewise, high levels of extraversion exhibit positive effects on social collaboration. Extraversion enhances interactions between team members, which is generally considered beneficial for overall group performance. The sociability and talkativeness of extroverts tend to support information exchange and interactions between group members [24,25] and create positive group climates in which individuals feel like they can express themselves [26]. Despite research identifying limitations in the extent to which extraversion contributes to group performance, for instance, that too many extraverts in a team can hinder group performance due to distractions resulting from social interaction [27], or social dominance [23], extraversion can be seen as a positive predictor overall. Finally, a high level of agreeableness is found among trusting, cooperative, and empathetic individuals, and facilitates interpersonal attraction, cooperation, smooth conflict resolution, open communication, and information seeking in groups (for more details, see [26]). Therefore, groups with lower levels of agreeableness and lower levels of tolerance, friendliness, helpfulness, and non-competitiveness tend to also have lower performance [24,26].

### 3.2. Personality and Cognitive (Problem Solving) Abilities

Cognitive problem solving accounts for the cognitive component of the conjoint construct of CPS. As problem solving can be seen as a facet of cognitive ability [28], existing findings on the relation between cognitive measures and personality can be helpful in forming hypotheses on the role of personality in CPS. A body of research and meta-analyses have identified consistent interrelations between personality traits and diverse cognitive tests assessing different aspects of cognitive ability (e.g., fluid and crystallized intelligence, reasoning, and memory), despite the longstanding debate on the degree to which personality and cognitive ability are inseparable or independent constructs [29,30]. Investment and developmental theories, such as the lifelong learning and investment model (PPIK) by Ackermann [11], ground relations between cognitive ability and affective traits in their interdependent development during childhood and adolescence, which ultimately creates patterns of abilities, skills, personality, and interests [12]. The PPIK theory integrates intelligence-as-process, personality, interest, and intelligence-as-knowledge, and therefore grounds ability levels and personality dispositions as determinates of success in particular task domains (e.g., science or mathematics) [13].

Personality generally accounts for approximately 5% to 10% of the variance in cognitive performance [31]. Hereby, the most consistent relations with cognitive ability have been identified for openness to experience and neuroticism. Openness to experience has been found to be a positive predictor of cognitive ability [32], as higher levels of openness to experience seem to induce stronger intellectual curiosity, interest, and engagement in cognitively stimulating tasks, which support the development of general cognitive ability [9,33]. Particularly in students, higher levels of openness to experience tend to be associated with general learning motivation [34] and critical thinking [35], positively affecting students’ academic performance in the form of grades in languages (i.e., German and French), and intelligence assessed via five subtests (i.e., figural thinking, reasoning, numerical, and arithmetical thinking), as found in PISA 2009 longitudinal data [36].

In direct contrast to this, neuroticism is a negative predictor of cognitive ability and problem solving. Neuroticism has been found to induce negative thoughts and test anxiety in testing conditions [12] and to decrease students’ general level of interest, which can lead to lower cognitive ability of attainment and skills [12] as well as dysfunctional thought processes, such as overgeneralizing or dependence on others [37]. Neuroticism also seems to impede students’ academic performance, for shifting students’ focus to emotional states and self-talk rather than academic task performance [38]. With regard to problem solving, neuroticism was negatively related to knowledge acquisition and application among students in virtual problem solving tasks [10].

Less consistent relations have been identified between cognitive ability and the three remaining Big Five traits of conscientiousness, extraversion, and agreeableness. Conscientiousness has sometimes been found to be negatively associated with cognitive ability and problem solving [10]. However, this is highly controversial, as conscientiousness has been found to be positively correlated with work-related outcomes (for possible reasons for this contradiction, see [30]). In student populations, conscientiousness is positively correlated with academic performance at school [39], particularly in mathematics, German, and French, and intelligence [36]. Similarly, inconsistent results have been found for the link between extraversion and cognitive ability. Extraversion has been identified as a positive predictor for diverse intelligence categories [40], including verbal learning, conditioning, and short and long-term memory recall [41]; however, it has also been found to be a negative predictor for performance in cognitive tasks [42,43]. For example, extraverted students seem to perform better academically due to higher energy levels and desires to learn [38] but also perform lower due to distraction resulting from socializing instead of studying [39]. Chamorro-Premuzic and Furnham [44] argue that a high level of extraversion supports academic performance during elementary school up to an age of approximately 12 years, but hinders it during secondary education. Likewise, inconsistent results have been obtained for the connection between agreeableness and cognitive ability. Only some facets of agreeableness have exhibited effects on cognitive ability, including a negative effect for aggressiveness [13], and positive effects for emotional perception and facilitation [45]. Findings about the link between agreeableness and academic performance are also consistently insignificant [46].

## 4. Purpose of This Study and Hypotheses

Despite the increasing relevance of CPS as a 21st century skill in students, and its integration into governmental education programs, such as PISA 2015 [14], there is little empirical evidence on how CPS is related to other constructs, its antecedents, or how it can be generally predicted. However, an existing body of research has identified the Big Five as predictors of social collaboration and cognitive problem solving. Considering that CPS is a conjoint construct of social collaboration and cognitive problem solving, the Big Five are expected to also play a role in CPS. Nevertheless, existing findings investigate either the personality–social collaboration link or the personality–cognitive ability link separately, and there is no research on the role of the Big Five in the conjoint construct of CPS. To overcome this, this study investigates the role of the Big Five in CPS and draws the following hypotheses from the separate bodies of literature on the personality–social collaboration link and the personality–cognitive ability link.

**H1:** The Big Five personality traits of openness to experience, conscientiousness, extraversion, and agreeableness will be positively associated with CPS.

**H2:** The Big Five personality trait of neuroticism will be negatively associated with CPS.

Existing findings on the relationship between cognitive ability and personality are consistent (see Section 3.2. Personality and Cognitive (Problem Solving) Abilities). To ensure that CPS is not only assessing cognitive performance, we control for two cognitive indicators in a second step of our analysis: Reasoning, due to its strong association with problem solving, and reading, as the PISA 2015 CPS units have a strong reading load. We hypothesize that the association between personality and CPS is distinct and therefore control for reasoning and reading performance in an additional step.

**H3:** The association between the Big Five personality traits on CPS will remain constant after controlling for reasoning and reading.

## 5. Method

### 5.1. Sample

A total of 748 students participated voluntarily in the study. Students were ninth and tenth graders at seven different secondary grammar schools in Germany. Grammar schools are the academically most demanding track in the German school system (for more information, please see [47]). Students without signed informed consent forms, or who had participated in pilot studies (*N* = 71) were excluded from the sample. We also dealt with missing data by design (*N* = 192) which had to be excluded from the final sample. The final convenience sample consisted of 483 participants (*M* = 15.80, *SD* = 0.65, 59.0% identified as female). Students received individual feedback on their results if requested.

### 5.2. Measures and Procedure

Collaborative Problem Solving (CPS). CPS was assessed using four of the seven original German PISA 2015 tasks we received from the OECD. As illustrated in Section 2, the PISA 2015 CPS tasks required students to collaborate with agents by exchanging predefined messages in a chat box, and performing actions (e.g., drag and drop) within a simulated problem in a task space (Figure 1). Writing directly to collaboration partners (so-called open chat communication) was not possible. Scoring was equivalent to the original PISA 2015 scoring, with one (or in rare cases, two) of the offered predefined messages, which represented a specific CPS skill, scored as the correct answer. All four tasks consisted of several consecutive task sections all within the same problem scenario; however, each task section was scored separately to allow for ongoing improvement throughout the units (for further information, please see [14]). In the tasks, students were given full credit (1 or in rare cases 2 points) for selecting the correct predefined messages and performing specific actions (e.g., correct drag and drop actions) in the problem space. Otherwise participants received no credit (0 points). To reduce the number of parameter estimations, we reduced the PISA 2015 CPS tasks into sum parcels [48]. Items were summed up to overall CPS scores for each CPS task.

Personality. The German version of the Neo Five-Factor Inventory (NEO-FFI) [49] was used to assess personality in a self-report format. The questionnaire consisted of a total of 60 items capturing the five personality traits of openness to experience, neuroticism, conscientiousness, extraversion, and agreeableness. Each trait was measured with 12 items on a 5-point Likert scale from 0 (*strongly disagree*) to 4 (*strongly agree*). Negatively phrased items were reversed. In accordance with the test manual, sums scores were calculated for each trait and divided by the number of answered questions. The resulting values reflected the strength of each personality trait.

Reasoning. The Intelligence Structure Test-Screening (IST-Screening) [50] was used to assess students’ reasoning skills. According to the IST-Screening manual, the test encompasses three subtests for numerical intelligence, verbal intelligence, and figural intelligence [50]. These subtests assessed each aspect of intelligence via number series, verbal analogies, and figure selection, and the overall test lasted approximately 30 minutes. Items were scored dichotomously such that students received full credit (1) or no credit (0) for each individual task. In accordance with the test manual, the sum of points was used to reflect the overall reasoning score.

Reading. Reading competency was assessed via one published paper-based PISA 2009 reading task “mobile phone security” (the German tasks can be viewed under http://www.men.public.lu/fr/themes-transversaux/qualite-scolaire/pilotage-monitoring/programme-international-pisa/). The task first had students read text material about a real-life situation (for example, information on mobile phone security). The task consisted of four items. The task included multiple-choice and open-response items. In accordance with the PISA 2009 coding guidelines, students received full credit (1) for selecting the designated correct answer in multiple-choice items, and no credit (0) otherwise [15]. In open response tasks, students were required to construct their own responses, for which they received full credit (1) for responding correctly, and no credit (0) otherwise [15]. Notably, open response tasks required their scoring by external independent coders following the PISA 2009 coding guidelines. Items were scored dichotomously such that students received full credit (1) or no credit (0) for individual tasks. In accordance with the PISA 2009 coding guidelines, an overall sum score was calculated by adding up the subscores on the task.

Procedure. The Luxembourgish National Commission for Data Protection, and the Education Ministries of Rhineland-Palatinate and Hesse approved the data collection in schools. Schools were recruited over email and participating classes received a financial reward in form of a donation of 160 Euro per class. Two trained test administrators assessed students over the course of one full school day (approximately 4.5 h) during regular class time, following a standardized assessment procedure for each class. Students completed several computer-based and paper-based performance tests and self-report questionnaires, from which the CPS, reasoning, and reading tests as well as the personality questionnaire are relevant to this study. At the beginning of each test day, students completed four original PISA 2015 CPS tasks individually over approximately two hours on MacbookPro laptops that were randomly assigned to students (i.e., students drew laptop numbers). In the PISA 2015 CPS tasks, students were required to solve virtual problem scenarios in collaboration with a minimum of one and a maximum of three computer-simulated agents. Students’ CPS performance was saved locally in the form of log files (i.e., files that contain users’ computer actions). After the PISA 2015 tasks, the paper-based performance tests and questionnaires were completed. To avoid cognitive fatigue in students over time during long test sessions [51], regular breaks were held in between performance tests and questionnaires.

### 5.3. Statistical Approach

We conducted latent regression analysis using structural equation modeling (SEM) in Mplus Version 7.0 [52] to test the association between the individual Big Five personality traits (i.e., openness to experience, neuroticism, conscientiousness, extraversion, and agreeableness) on CPS (H1 and H2; Model A), also when controlling for reasoning and reading (H3; Model B). Both models included missing data by design, as some students completed particular PISA 2015 CPS tasks in a reformatted version for the purpose of another study. Because this study was designed to work with original PISA 2015 CPS tasks, these students’ values were replaced with missing values. In SEM, we chose the maximum likelihood estimator for robust standard errors and fit statistics against normality violations (MLR) for our model due to the nature of our continuous variables. We applied the “type is complex” command in MPlus7, for which a minimum of 20 classes is required, to include the hierarchical structure of our nested data (i.e., students nested in 32 different classes) in Models A and B and adjust standard errors. Model fit was evaluated according to standard fit indices and cut-off values, namely the comparative fit index (CFI, cut-off *CFI* > .95 for good fit), Tucker–Lewis index (TLI, cut-off *TLI* > .95 for good fit), root-mean-square-error-of-approximation (RMSEA, cut-off *RMSEA* < .05 for good fit), and standardized-root-mean-square-residual (SRMR, cut-off *SRMR* < .05 for good fit). In a final step, we controlled for reasoning and reading competency, assessed using the IST-Screening and PISA 2009 reading tasks, to identify whether the association between personality and the Big Five is distinct (H3). For this, we used a technique of using residuals to control for common variance between CPS and reading and reasoning, respectively [53]. Hereby, we regressed CPS on reasoning and reading performance, and subsequently included only the residual of CPS as a criterion for the Big Five personality traits (Model B).

## 6. Results

### 6.1. Measurement Model and Descriptive Statistics

The CPS measurement model with four indicators (the four CPS tasks) exhibited tenable model fit (*χ*^2^ = 80.92, *df* = 4, *p* > .05; *CFI* = 1.00, *TLI* = 1.00, *RMSEA* = .000, *SRMR* = .014, *N* = 483). Table 2 provides details on the manifest and latent correlations, means, and standard deviations for the measures in this study.

### 6.2. H1 and H2 (Relation between Personality and CPS)

**Figure 2:**
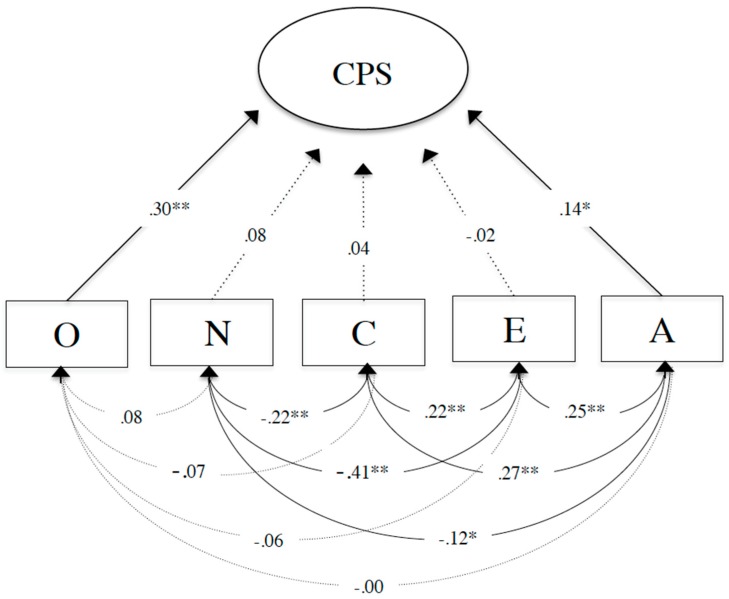
Structural equation model (Model A) presenting the associations between the self-reported Big Five personality traits of openness to experience (O), neuroticism (N), conscientiousness (C), extraversion (E), agreeableness (A), and CPS. Significant standardized parameter estimates are significant at the ** *p* < .01 or * *p* < .05 level and presented as solid black arrows. Non-significant standardized parameter estimates are presented as dotted arrows.

To test the association between personality and CPS performance, we included the Big Five personality traits of openness to experience, neuroticism, conscientiousness, extraversion, and agreeableness as predictors, and CPS performance as criterion in the first model (Model A). The Big Five personality traits were modeled as manifest sum scores, whereas CPS was modeled as a latent factor with four indicators. Model fit was tenable (Model A: *χ^2^* = 20.926, *df* = 17, *p* > .05, *CFI* = .980, *TLI* = .970, *RMSEA* = .022, *SRMR* = .038, *N* = 483). As presented in Figure 2, we found associations between the Big Five personality traits and CPS performance. Hereby, the Big Five traits of openness to experience (*ß* = .30, *SE* = .06, *p* < .01) and agreeableness (*ß* = .14, *SE* = .06, *p* < .05) were positively associated with CPS performance. As expected in H1, students with higher levels of openness to experience and agreeableness achieved higher performance scores than students with lower levels of openness to experience and agreeableness. Conscientiousness (*ß* = .04, *SE* = .07, *p* > .05) and extraversion (*ß* = −.02, *SE* = .06, *p* > .05) were not significantly associated with CPS. In contrast to our expectation in H2, neuroticism (*ß* = .08, *SE* = .05, *p* > .05) exhibited a slightly positive but non-significant relation to CPS. The results for H1 confirmed our expectations to the extent that the two Big Five personality traits openness to experience and agreeableness were positive predictors of CPS performance.

### 6.3. H3 (Relation between Personality and CPS Controlling for Reasoning and Reading)

**Figure 3:**
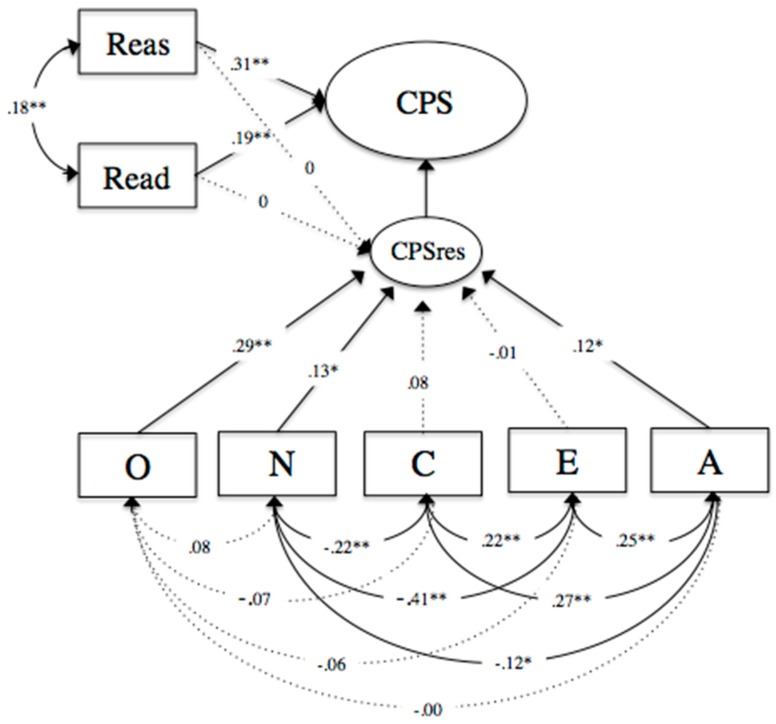
Structural equation model (Model B) presenting the associations between the self-reported Big Five personality traits of openness to experience (O), neuroticism (N), conscientiousness (C), extraversion (E), agreeableness (A), and CPS, controlling for reasoning (Reas) and reading (Read). CPSres represents the disturbance term for CPS. Correlations were allowed between all manifest variables. Significant standardized parameter estimates are significant at the ** *p* < .01 or * *p* < .05 level.

In a next step, we controlled for reasoning and reading to identify whether the association between personality and CPS is distinct and therefore control for reasoning and reading performance in CPS. Model fit was tenable (Model B: *χ*^2^ = 36.346, *df* = 23, *p* > .05, *CFI* = .951, *TLI* = .927, *RMSEA* = .035, *SRMR* = .038, *N* = 483). Equivalent to Model A, the Big Five traits of openness to experience (*ß* = .29, *SE* = .05, *p* < .01) and agreeableness (*ß* = .12, *SE* = .06, *p* < .05) positively predicted CPS performance. Similarly to Model A, the traits of conscientiousness (*ß* = .08, *SE* = .07, *p* > .05) and extraversion (*ß* = −.00, *SE* = .06, *p* > .05) exhibited non-significant associations with CPS. In contrast, neuroticism (*ß* = .13, *SE* = .04, *p* < .01) was a significant positive predictor for CPS (see Figure 3).

## 7. Discussion

The aim of the current study was to investigate whether and to what extent personality can predict CPS. For this, the study applied SEM to test the associations of the Big Five personality traits, that is, openness to experience, neuroticism, conscientiousness, extraversion, and agreeableness, with CPS. As expected, SEM identified positive associations between the Big Five personality traits of openness to experience and agreeableness and CPS performance. These results remained mostly stable when controlling for reasoning and reading performance.

### 7.1. Openness to Experience and Agreeableness Predict CPS beyond Reasoning and Reading

In our analyses, the Big Five trait of openness to experience remained the strongest predictor of CPS, as expected from the academic literature showing a link between openness to experience and cognitive ability as well as social collaboration. We assume that higher levels of openness to experience in students induced stronger engagement with the cognitive problem solving tasks and increased information exchange with the computer agents in the PISA 2015 CPS tasks [9,33]. This might explain why students with higher levels of openness to experience achieved higher CPS performance. These results are in line with the PPIK model [11], which stipulates a positive relation between openness to experience and intelligence [43] due to stronger intellectual curiosity, interest, and engagement in cognitively stimulating tasks, which support the development of general cognitive ability [9,33].

In addition to openness to experience, agreeableness also predicted higher CPS performance in students. It is plausible that higher levels of agreeableness induced more cooperation, conflict resolution, and communication with the computer agents, all of which are a relevant part of the PISA 2015’s understanding of CPS. That is, considering that the computer agents in PISA 2015 CPS tasks deliberately create disagreements in order to test how students communicate in such situations and resolve the problem [14], agreeable students seem to have been able to resolve these situations more often, which in turn led to higher CPS performance. In addition, PISA 2015 weighted collaboration actions higher than problem solving actions in its scoring methodology, which may have further supported the role of agreeableness in CPS performance. These results on openness to experience and agreeableness remained stable when controlling for reasoning and reading. The correlations between reasoning and reading and CPS were relatively strong (see Table 2), and controlling for the variance of reasoning and reading in CPS ensured that the associations between personality and CPS were distinct. Also, controlling for reasoning in CPS extracted aspects of reasoning-based problem solving and emphasized the social collaboration aspects of the conjoint PISA 2015 CPS construct CPS. As openness to experience and agreeableness remained stable predictors for CPS, we can say that these predictors are essential for social interaction in CPS environments.

Further, our findings do not support the negative association between neuroticism and cognitive ability and social collaboration (e.g., [10]). Neuroticism showed a weak positive association with CPS, which was significant when controlling for reasoning and reading performance. Obviously, the social collaboration in the CPS tasks was artificial as all collaboration partners were agents, which was openly disclosed to the participants. It therefore seems plausible that social anxiety or the fear of being evaluated by the collaborators would not limit participants’ performance. The positive association between neuroticism and CPS still remains surprising. It is possible that, once the fear of being evaluated was removed, students with higher neuroticism actually benefitted from their greater attention to the social interaction. This remains purely speculative, though, and needs to be corroborated in future studies.

The correlations between openness and the other dimensions of personality were considerably lower than to be expected based on previous meta-analyses [54]. This may be due to the relatively low average age of our sample as other studies also reported small to zero correlations between openness and other dimensions of personality in samples of children and young adults [55,56].

Overall, our results did not contradict the PPIK theory to the extent that personality dispositions act as determinates for success in particular task domains, and that cognitive ability cannot be fully understood without integrating personality differences. In our case, openness to experience acted as a determinate for success in CPS performance even though it was not significantly related to reasoning. Going a step further, investment theories, such as the PPIK theory, argue that patterns of personality, interests, and cognitive ability become increasingly consistent with age as interests steer individuals’ attention to life experiences (for an alternative view, refer to [9]). In addition, intra-individual differences in cognitive ability and non-ability trait determinants develop mostly during the school years and stabilize at the end of high school [57]. Therefore, our results should be generalizable to adults, as the participants in our study were at the end of compulsory education (age *M* = 15.80). Finally, looking at personality at the facet level, rather than the domain level, might lead to stronger and even more interesting findings [58].

### 7.2. Limitations

To the best of our knowledge, this study remains the only study on the role of personality in CPS performance. As this study entails limitations, future empirical studies that address these should be conducted to establish knowledge regarding the relatively new construct of CPS. First, this study assessed a pre-specified sample of adolescents who were of the same age as the PISA samples. Despite our assumption that our findings can be generalized to adults on the basis of the PPIK theory, further studies should test this using adult samples. Furthermore, we employed the PISA 2015 understanding of CPS as a conjoint construct encompassing social collaboration and cognitive problem solving skills. The overall correlations found between CPS and personality were rather low, though. As personality and cognitive ability combined could not explain all the variance in CPS, the chosen definition of CPS may not be adequate. Defining and employing CPS as a non-conjoint construct may allow the role of personality within each specific component to be identified (see also [9]). Moreover, reading was assessed with only one PISA 2009 task, which showed an unacceptably low internal consistency. The effect sizes for reading are therefore likely to be underestimated by our models. Future studies should either use the complete PISA 2009 reading scale in assessing reading, or use a different empirically established assessment measure for reading.

### 7.3. Conclusions and Future Outlook

Despite the increasing recognition of CPS solving as an important 21st century skill by governmental and educational leaders, particularly for computer-based environments, scientific research on CPS has been scarce, lagging far behind the political and educational relevance of CPS. There is scant empirical evidence on how CPS relates to other constructs, its antecedents, or how it can be generally predicted. This study identified whether, and which of the Big Five personality traits predict CPS in PISA 2015 CPS tasks. We found that openness to experience and agreeableness were positive predictors for CPS performance, even after controlling for reasoning and reading performance. In other words, students who reported higher levels of openness to experience and agreeableness achieved higher CPS scores assessed using the PISA 2015 computer-based approach. These results contribute important information for the development of educational assessments and educational interventions for CPS. For example, intervention strategies for students with low levels of openness to experience or agreeableness can be developed to adequately prepare students for realistic future environments, including computer-based environments. In addition to this study’s theoretical and practical contribution to CPS research, it also provides the first practical indications of students’ PISA 2015 CPS performance results that have been published in a special report by [6] just recently.

## Figures and Tables

**Table 1:** The 12-Cells Matrix illustrating the 12 collaborative problem solving (CPS) skills in the Programme for International Student Assessment (PISA) 2015 Assessment. Drawn from the Organisation for Economic Co-operation and Development (OECD) CPS Draft Report in PISA 2015 [6].

	(1) Establishing and Maintaining Shared Understanding	(2) Taking Appropriate Action to Solve the Problem	(3) Establishing and Maintaining Team Organization
(A) Exploring and Understanding	(A1) Discovering the perspectives and abilities of team members	(A2) Discovering the type of collaborative interaction needed to solve the problem, along with goals	(A3) Understanding roles to solve problem
(B) Representing and Formulating	(B1) Building a shared representation and negotiating the meaning of the problem (common ground)	(B2) Identifying and describing tasks to be completed	(B3) Describe roles and team organization (communication protocol/rules of engagement)
(C) Planning and Executing	(C1) Communicating with team members about the actions to be / being performed	(C2) Enacting plans	(C3) Following rules of engagement (e.g., prompting other team members to perform their tasks)
(D) Monitoring and Reflecting	(D1) Monitoring and repairing the shared understanding	(D2) Monitoring results of actions and evaluating success in solving the problem	(D3) Monitoring, providing feedback, and adapting the team organization and roles

**Table 2:** Manifest (above the diagonal) and latent (below the diagonal) correlations for the Big Five personality traits of openness to experience (*O*), neuroticism (*N*), conscientiousness (*C*), extraversion (*E*), agreeableness (*A*), collaborative problem solving (*CPS*), reading performance (*Read*), and reasoning (*Reas*), as well as McDonald’s Omega values (*ω*).

	*M*	*SD*	*O*	*N*	*C*	*E*	*A*	*CPS*	*Read*	*Reas*	*ω*
O	2.20	0.55	-	.08	−.07	−.06	−.01	.30 **	.15 **	.05	.97
N	1.58	0.67	.09	-	−.22 **	−.41 **	−.12 *	.09	.01	−.10 *	.99
C	2.61	0.62	−.06	−.20	-	.22 **	.27 **	.05	−.02	−.05	.99
E	2.48	0.55	−.06	−.42	.21	-	.25 **	−.04	−.03	−.02	.98
A	2.57	0.51	.01	−.12	.27	.25	-	.14 *	.08 *	.02	.97
CPS	0	1	.30	.08	.03	−.03	.15	-	.30 **	.34 **	.96
Read	2.58	0.98	.15	.08	.02	−.04	.10	.27	-	.21 **	.51
Reas	45.24	5.63	.03	−.13	−.11	−.00	.01	.27	.09	-	.91

*Note.* The Big Five personality traits (O/N/C/E/A), reading performance (Read), and reasoning (Reas) were manifest variables and CPS was a latent variable. Total *N* = 483. *M* = mean. *SD* = standard deviation. * *p* < .05. ** *p* < .01.
